# Association between Fluorescent Advanced Glycation End-Products and Vascular Complications in Type 2 Diabetic Patients

**DOI:** 10.1155/2017/7989180

**Published:** 2017-12-06

**Authors:** Alexis Guerin-Dubourg, Maxime Cournot, Cynthia Planesse, Xavier Debussche, Olivier Meilhac, Philippe Rondeau, Emmanuel Bourdon

**Affiliations:** ^1^Université de La Réunion, INSERM, UMR 1188 Diabète Athérothrombose Thérapies Réunion Océan Indien (DéTROI), Saint-Denis de La Réunion, France; ^2^Centre Hospitalier Gabriel Martin, Saint-Paul, France; ^3^Centre Hospitalier Universitaire de La Réunion, Saint Denis, France

## Abstract

**Objectives:**

Diabetes is a major health problem associated with hyperglycemia and chronically increased oxidative stress and enhanced formation of advanced glycation end-products (AGEs). The aim of this study was to determine whether oxidative plasma biomarkers in diabetic patients could be evidenced and associated with vascular complications.

**Methods:**

Oxidative stress biomarkers such as thiols, ischemia-modified albumin (IMA), glycated albumin (GA), fructosamine, and AGEs were measured in 75 patients with poorly controlled type 2 diabetes (HbA1c > 7.5%) with (44) or without (31) vascular disease and in 31 nondiabetic controls.

**Results:**

Most biomarkers of oxidation and glycation were significantly increased in diabetic patients in comparison with nondiabetics. Fructosamines, GA, IMA, and AGEs were positively correlated and levels of fluorescent AGEs were significantly increased in the plasma from patients presenting vascular complication.

**Conclusions:**

These results bring new evidence for the potential interest of glycated albumin, oxidative stress, and glycoxidation parameters in the monitoring of type 2 diabetic patients. Furthermore, it emphasizes fluorescent AGEs as a putative indicator for vascular event prediction in diabetic patients.

## 1. Introduction

Type 2 diabetes mellitus (T2DM) is a metabolic disease which has reached pandemic proportions and is considered as one of the world's most important causes of healthcare expenditure, disability, and mortality. Enhanced morbidity in diabetes is mainly associated with micro- and macrovascular complications [1]. Indeed, T2DM could double the risk of developing cardiovascular diseases (CVD) including stroke and myocardial infarction, which represent the leading cause of mortality in western and developing countries [2]. In Réunion Island, a French overseas territory, diabetes prevalence is particularly high (>20% of adult people) and is associated with a higher vascular disease-related mortality (×2) when compared to mainland France [3].

Glycation is clearly identified as a deleterious phenomenon in diabetic complications and the association with oxidative stress seems to be deleterious too [4, 5]. One of the underlying features of hyperglycaemia is the excessive nonenzymatic glycation of the two main circulating proteins: haemoglobin and albumin. This chemical process consists in a complex cascade of reactions between glucose or derivatives with proteins, yielding a heterogeneous class of compounds termed advanced glycation end-products (AGE) [6]. Glycation process, in conjunction with oxidative stress named “glycoxidation” can cause structural and functional impairments of plasma proteins in particular albumin [7–9] and was involved in pathophysiological mechanism of vascular diseases in T2DM [10, 11]. Many clinical studies have suggested glycated albumin (GA) and ischemia-modified albumin (IMA) as additional and/or alternative plasma biomarkers of poor glycemic control, redox state, and glycation levels in diabetic subjects [12–14]. Similarly, the redox state of plasma thiols could also be used as a marker for several pathologies such as early atherosclerosis and liver disease [15, 16]. Considered as an important intracellular redox regulator with a potential role in stress signalling, plasma thiols levels could be suggested as an oxidative stress biomarker [17].

Identification of novel vascular risk biomarkers as potential indicators for CVD and stroke prevention and intervention in T2DM remains highly warranted. The increase of plasma levels of glycoxidation and oxidative stress markers could be associated with an increase in risk of developing vascular diseases [18, 19]. Both glycation and oxidative stress may produce biomarkers (AGE and IMA for instance) that could be used to predict vascular disease [10, 20].

The aim of this study was to determine whether oxidative plasma biomarkers in diabetic patients could be evidenced and associated with vascular complications related to diabetic micro- and macroangiopathy.

## 2. Materials and Methods

### 2.1. Subjects

The Alb-Ox ERMIES is an ancillary, pilot study of ERMIES (NCT01425866), in which we performed a case/case/control analysis of 106 patients. The diabetic cases considered here are from the ERMIES study (structured self-management education maintained over two years in insufficiently controlled type 2 diabetic patients) whereas controls come from patient samples of biochemistry unit of local hospital (Saint-Denis, La Réunion). Blood was sampled on EDTA tubes (BD Vacutainer).

Diabetic subjects from ERMIES are T2DM patients from La Réunion aged over 18 years with high HbA1c levels (>7.5% for more than three months) enrolled between October 2011 and April 2014 (D, *n* = 75) [21]. They were treated with OHA and/or GLP-1 (oral hypoglycaemic agents/Glucagon-like peptide 1) analogue and/or insulin therapy for at least one year and they followed a stable drug treatment regimen for at least 3 months. All patients with initial severe complications including ischemic or proliferative diabetic retinopathy, severe chronic renal failure, active coronary artery insufficiency, diabetic foot lesion, or cancer were excluded. In the control group (ND, *n* = 31), patients were over 18 years old and had no symptom of uncontrolled diabetes with low HbA1c levels (<6.0%). None of the control subjects was under antidiabetic medication. All eligible subjects provided information on the history of their pathology (previous vascular events, medication, etc.) and underwent a medical examination aimed at assessing their micro- or macroangiopathic complications. All biological data related to the Alb-Ox ERMIES study are given in Table 1.

The pool of diabetic patients was divided into two groups according to the degree of vascular disease assessed in the ERMIES study entry (medical history and clinical review). The main vascular complications (diabetic micro- and/or macroangiopathy) listed in this study are nephropathy, retinopathy, peripheral neuropathy, diabetic foot for microangiopathy, acute coronary syndrome, coronary angioplasty, stable angina, and ischemic stroke for macroangiopathy. Vascular complications were evaluated during the open-label lead in phase (OL LI) following a clinical and biological examination. Type 2 diabetes group included *n* = 44 subjects with a history of vascular disease (VD) and *n* = 31 subject without any vascular complication before the study or at the time of the initial enrolment (NVD).

### 2.2. Determination of HbA1C Levels

Glycated haemoglobin levels were assessed on anticoagulated whole blood stored at 4°C less than 48 hours after collection in EDTA tubes (BD Vacutainer). HbA1c levels were measured by using a capillary electrophoresis method performed on automate Capillary Flex (SEBIA laboratory).

### 2.3. Biochemical Analysis

Total proteins, albuminemia, fructosamine, triglyceride (TG), total cholesterol (CHOL), low-density lipoprotein (LDL), high-density lipoprotein (HDL), Apolipoprotein A1 (ApoA1), and Apolipoprotein B (ApoB) levels in plasma were determined using a clinical biochemistry automate Cobas C501 analyzer (Roche Diagnostic). Analyses were performed on EDTA-plasma within 6 hours after blood sampling. Total blood and plasma samples were stored at room temperature during these analyses. Then, plasma samples were stored at −80°C before additional biochemical tests.

### 2.4. Additional Biochemical Characterizations

Thiols group concentration in plasma proteins was measured by Ellman's assay using 5,5-dithiobis-(2-nitrobenzoic acid) (DTNB) [22] as previously described [23].

Glycated albumin and AGE levels were quantified in plasma by using a commercially available ELISA kits for detection of human GA and AGEs (Sunred Bio) according to the manufacturer's protocol. The AGE ELISA commercial kit quantifies preferentially nonfluorescent AGEs (including N(*ε*)-Carboxymethyllysine (CML) or N(*ε*)-Carboxyethyllysine (CEL)) relative to fluorescent AGEs such as Pentosidine or Vesperlysine [6].

The fluorescence intensity of AGEs in plasma was obtained at 380 nm excitation and 420 nm emission wavelengths using a Horiba FluoroMax®-4 spectrophotometer. The excitation and emission slits were equal to 5 and 10 nm, respectively. All protein samples were prepared at 1.5 mg/mL in 50 mM sodium phosphate buffer at pH 7.4. Fluorescent AGE (Fluo-AGE) (mainly Pentosidine, Vesperlysine, and Crossline) levels were expressed in arbitrary units.

The albumin-cobalt binding (ACB) test reported by Bar-Or et al. was originally designed to detect ischemia-modified albumin (IMA) in patients with myocardial ischemia [12, 24]. This assay based on the reduced binding affinity of human serum albumin for metal ions (Cobalt, Co^2+^) was applied here on human plasma samples, as previously described [25]. Preparations for the Co (II) albumin binding protocol consist in the addition of 20 *μ*l of samples (0.15 mM) to 15 *μ*l of a 0.2% cobalt chloride solution, followed by vigorous mixing and 15-min incubation at 37°C. Dithiothreitol (DTT, 20 *μ*l of a 1.5 g/l solution) was then added and mixed. After incubation for 2 minutes, 20 *μ*l of 0.9 M NaCl solution was added. The absorbance (*D*1) was read at 470 nm using a microplate reader. The corresponding blank was prepared similarly without DTT and read at the same wavelength (*D*0). The IMA index was expressed as absorbance Δ*D* = *D*1 − *D*0 which represents the residual unbounded Co^2+^ amount and then normalized to plasma albumin concentration according to the following formula:

(1) $$\begin{array}{c} \frac{IMA}{ALB}=\Delta D*\left[\;ALB\;\right]. \end{array}$$

In the previous formula, [ALB] corresponds to serum albumin concentration (g/L).

### 2.5. Statistical Analysis

Means ± SD or standard error to the mean (SEM) were presented for normally distributed variables and medians (25th–75th percentile) for nonnormally distributed variables. Statistical significance was determined using Student's *t*-test or the Mann–Whitney test. For regression analyses, we log-transformed all biomarker concentrations with skewed distributions. Spearman's correlations were used to assess the relationships between biomarkers. Univariate and multivariate logistic models were considered to assess the association between AGE levels and the presence of microangiopathy after adjustment for potential confounders. The multivariate model was adjusted for cardiovascular risk factors: age, sex, and body mass index. *P* values < 0.05 were considered statistically significant. Analyses were conducted using STATA 13 software (STATA corp, Tex, USA).

## 3. Results

**Figure 1:**
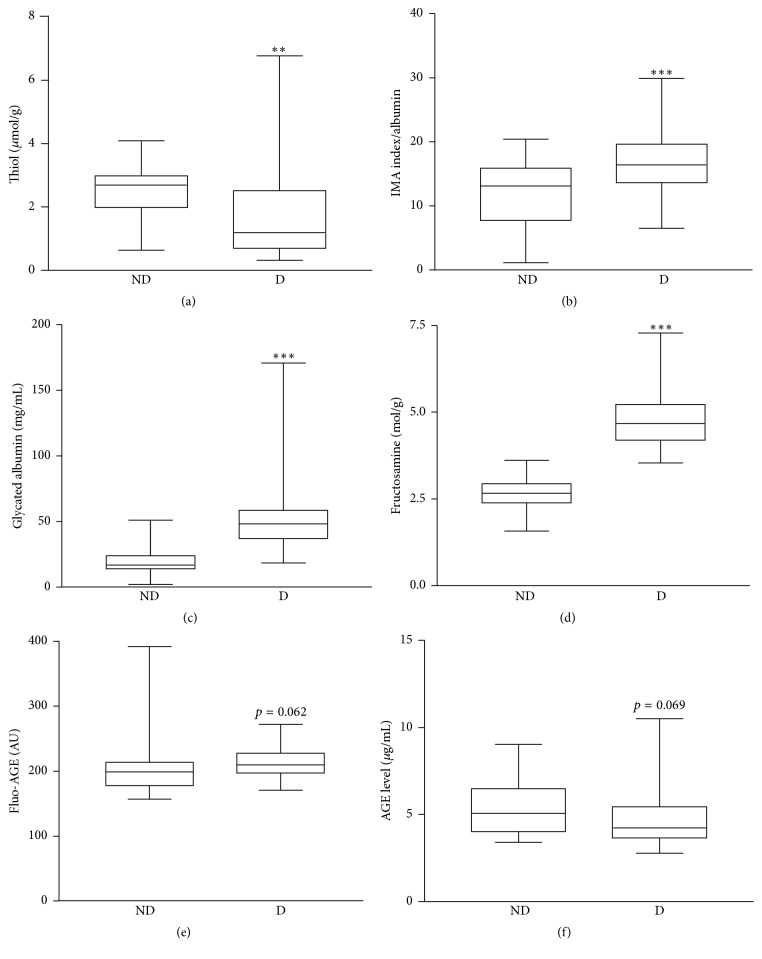
Box-plot distribution of biomarker values in diabetic (D) and nondiabetic (ND) groups. (a) Thiol levels (*μ*mol/g protein); (b) IMA index (AU/g albumin); (c) Glycated albumin levels (mg/mL); (d) Fructosamine levels (mol/g protein); (e) Fluo-AGE levels (AU/g); (f) AGE levels (*μ*g/mL). The horizontal bars in the box represent the median; the box boundaries represent the 25th and 75th percentile values. ^*∗∗∗*^*P* < 0.001 and ^*∗∗*^*P* < 0.01 indicate a significant difference versus ND group.

The clinical and biochemical characteristics of participants are reported in Table 1. Type 2 diabetes group included 31 obese patients (BMI > 30) including those who presented severe obesity (BMI > 40, *n* = 5). Patients with T2DM had significantly higher levels of albumin, fructosamine, and HbA1c than controls. In contrast, for others biochemical parameters (triglycerides, cholesterol, and apolipoproteins) the differences did not reach statistical significance. As shown in Figure 1, several oxidation and glycation biomarkers were significantly higher in the diabetic group (D) as compared to the control group: IMA, GA, and fructosamine. Conversely, thiols levels were significantly lower in the diabetic group (1.04 ± 0.02) than in the control group (2.47 ± 0.03). Only nonsignificant variations could be observed for glycoxidation biomarkers; Fluo-AGE and AGE levels were, respectively, higher and lower in diabetic patients relative to controls.

As featured in Table 2, different correlations were established between biomarkers of oxidative stress (thiol, IMA), glycation (glycated albumin, fructosamine), and glycoxidation (Fluo-AGE and AGE). Significant correlations were established between these biomarkers: IMA index was found to be positively correlated with fructosamine (*r* = 0.430, *p* < 0.0001), GA (*r* = 0.302, *p* < 0.0001), and with Fluo-AGE (*r* = 0.219, *p* < 0.05) levels. IMA index does not appear to be correlated with AGE levels. As expected, a marked relationship was noticed between fructosamine and GA (*r* = 0.492, *p* < 0.0001) and to a lesser extent between AGE and GA (*r* = 0.399, *p* < 0.0001). However, there was no significant correlation between thiol levels and other markers.

**Figure 2:**
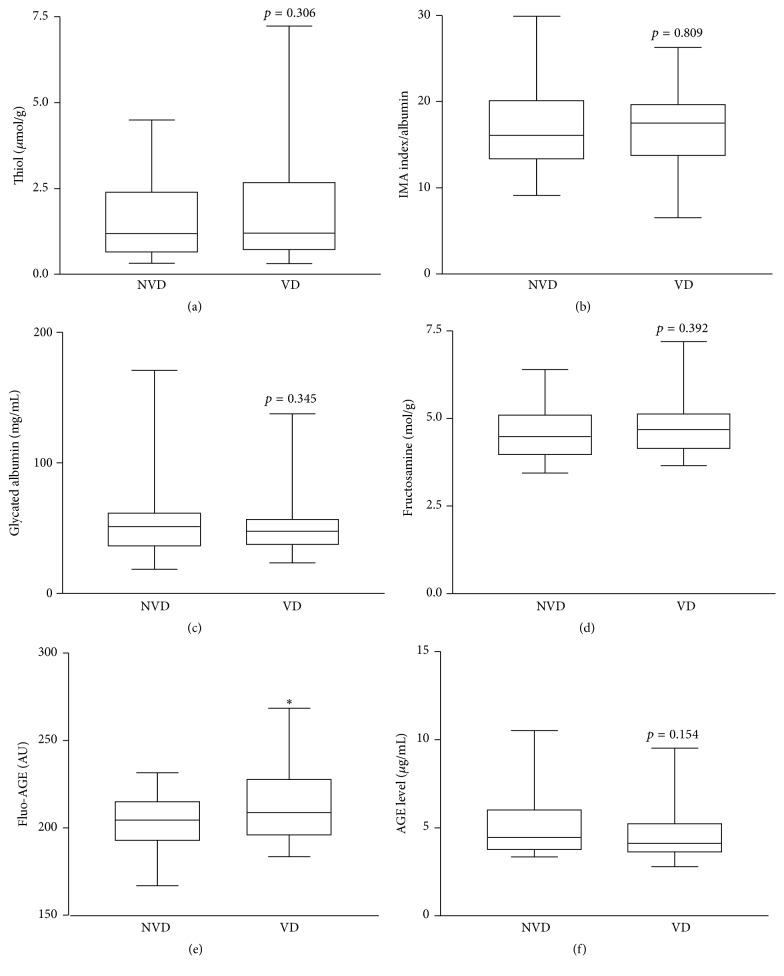
Box-plot distribution of biomarker values in the diabetic group with and without vascular disease groups (resp., VD and NVD). (a) Thiol levels (*μ*mol/g protein); (b) IMA index (AU/g albumin); (c) Glycated albumin levels (mg/mL); (d) Fructosamine levels (mol/g protein); (e) Fluo-AGE levels (AU/g); (f) AGE levels (*μ*g/mL). The horizontal bars in the box represent the median; the box boundaries represent the 25th and 75th percentile values. ^*∗*^*P* < 0.05 indicates a significant difference versus NVD group.

The clinical and biochemical characteristics of diabetic patients according to the history of vascular disease are summarized in Table 3. Forty-four diabetic subjects presented a vascular disease such as microangiopathy (*n* = 30), macroangiopathy (*n* = 3), or both complications (*n* = 11). The comparison between both groups did not show any significant difference in biological parameter levels. As for specific biomarkers of oxidative stress, glycation, and glycoxidation, only Fluo-AGE levels were found to be discriminatory between groups. Indeed, the Fluo-AGE levels were significantly higher in VD group than in NVD group (*p* < 0.05). However, the increases of IMA index (*p* = 0.809) and fructosamine levels (*p* = 0.392) in VD group were not statistically significant as shown in Figure 2.

The correlation coefficients analysis featured in Table 4 did not show any significant relationship between main biological parameters of oxidation and glycation. However and noteworthy enough, a significant correlation was established between glycated albumin and nonfluorescent AGE levels (*r* = 0.4312, *p* < 0.0001).

Because confounding factors such as sex, age, and BMI could influence both AGEs products and vascular complication occurrence, we performed a multivariate analysis (Table 5). Significant association between fluorescent AGE levels and personal history of microangiopathy was evidenced using univariate analyse. When adjusted for age, sex, and BMI, the association between fluorescent AGE levels and personal history of microangiopathy failed to reach significance (*p* = 0.07).

## 4. Discussion

Our study was designed to investigate a potential relationship between specific biomarkers of oxidation, glycation, and glycoxidation in diabetic patients and whether they could be associated with vascular complications related to diabetic microangiopathy and macroangiopathy. If some of these markers associated with diabetes were clearly correlated with one another (such as IMA with GA and fructosamine or GA with fructosamine), others (i.e thiols and AGEs) were independent. Biomarker of oxidation, early glycation, or advanced glycoxidation appeared independent when considering the sole diabetic group. In addition, these biochemical parameters provide nonredundant information to fructosamine and HbA1c assays.

T2DM is characterized by chronic hyperglycemia which can induce different molecular mechanisms involved in diabetes-associated metabolic disorders [26]. Through several pathways, hyperglycemia induces an increase of oxidative stress leading to an overproduction ROS and free radical species associated with an impairment of antioxidant defence systems [27]. The nonenzymatic glycation process occurring at higher rate in diabetic versus healthy subjects gives rise to the formation of early glycation products such as fructosamine which can evolve to AGEs [6]. In oxidative stress situations, these AGEs may result from oxidation reactions of early stage glycation products [28]. The overproduction of ROS contributes to structural, biochemical, and functional alteration of albumin resulting in a decrease of thiol levels associated with an increase of IMA index [9, 13]. In parallel, the nonenzymatic glycation process combined with oxidation reaction promotes the formation of early glycation products (fructosamine, Amadori products, or GA) and eventually of terminal products (AGEs) derived from albumin. The results presented here, indicating significant increases of IMA, GA, and fructosamine levels and decrease of plasmatic thiol level with type 2 diabetic patients, are in full accordance with the pathophysiological process occurring in a hyperglycemic context.

The absence of correlation between thiol and IMA levels is somehow surprising. Indeed, IMA index is related to the reduced binding capacity of HSA for Co^2+^ metal ions. It was already demonstrated that the structural integrity of HSA was essential for the binding between albumin and cobalt [13]. The alteration of plasma redox state in diabetic situation, characterized by a significant decrease in thiol levels, may have a direct impact on IMA levels. Compared to another study, the correlation between IMA and glycated albumin obtained here was also unexpected [29]. Indeed, this clinical study demonstrated that IMA was rather correlated with fasting glucose levels rather than with glycated albumin. In addition, IMA was also reported as an early marker of myocardial ischemia in patients after a percutaneous coronary intervention because IMA level variations were detected only few minutes after intervention and remain stable after few hours [30].

Among T2DM patients, glycated albumin was positively associated with AGE levels, reflecting that AGE formation is an early pathophysiological process involving glycated albumin. In addition, most of biomarkers displayed similar levels in patients with and without vascular disease. With a *p* < 0.05, the variation in fluorescent AGE level appeared to be detectable between both diabetic groups. This result appears to be in accordance with previous clinical study and fluorescent AGE could represent a relevant biomarker for the detection of vascular events in diabetic patients after validation in a broader clinical study. Indeed, a large scale clinical study has recently shown that the activation of the receptor for advanced glycation end-products (RAGE) by AGE is implicated in the development of diabetes complications [31].

AGEs are heterogeneous and complex structures, which may represent important mediators of diabetic vascular complications. AGEs notably induce alterations of both functional and mechanical properties of tissue via crosslinking intracellular and extracellular proteins [32–34]. In support, some studies have shown correlations between serum AGE levels and the development/severity of vascular disease [35]. Besides, others failed to established associations of some specific plasma AGEs with CVD in individuals with normal glucose metabolism or T2DM [36, 37].

Because of their heterogeneous structures, the contribution of each AGE molecule to the pathophysiology remains unknown [38]. Among AGEs, fluorescent ones are crosslinking structures such as Pentosidine and Crossline and known to contribute to the development of vascular complications [39]. In particular, several studies in diabetic or hemodialysis patients reported a correlation between serum Pentosidine levels and arterial wall stiffness, a recognized component in the determination of vascular risk [40, 41]. Another study, evaluating the prognostic value fluorescent AGEs in the context of acute coronary syndrome, reported the association of fluorescent AGE levels and the occurrence of cardiac events during the follow-up period [42].

Although an increased value of IMA was found in diabetic group when compared with nondiabetics, this oxidative stress marker displayed no significant variations among the diabetic group. Contradictory results were reported in other studies of T2DM patients with peripheral arterial disease or patients who had stroke [43, 44]. In these studies, IMA and other glycation markers such as HbA1c were assessed in diabetic patients with recent or progressive vascular events while in the present study all patients with progressive or recent serious vascular complications such as myocardial infarction or cerebral vascular accident were excluded. This could partly account for the lack of difference and this should be considered as a limitation in the study. The absence of significant variations of IMA index in our study could also be due to the limited sensitivity of this parameter at early stage of vascular events. Our study is original since all patients presented a serious long-standing T2DM (duration of diabetes greater than 10 years and HbA1c > 7.5%) always associated with overweight or obesity (BMI > 25). The use of IMA index as an alternative indicator for determining vascular risk and oxidative stress has generated numerous studies [43, 45, 46].

A number of factors may account for the lack of significant differences between the diabetics with and without vascular complications in our study. First of all, very few cases of macroangiopathy were observed in the VD group that consisted essentially in patients with diabetic microangiopathy. Secondly, the sample size of our study probably limited the statistical power. Biomarkers may accumulate in the plasma due to renal insufficiency. The absence of data concerning glomerular filtration rate or creatinemia of our patients represents a weakness of this present work. At last, a limitation of this work is the cross sectional design which did not allow investigation of causal mechanisms connecting glycoxidation and vascular complications.

In order to evaluate the different biomarkers of oxidative stress and glycation as potential predictors of vascular disease in T2DM patients, a broad cohort study would be needed including the collection of biological samples and the vascular disease assessment. The carotid intima media thickness (IMT) determined by echo tracking [44] or the arterial stiffness estimation by pulse wave velocity (PWV) [47] could be considered as substitute criteria of vascular disease burden reflecting atherosclerosis.

Further studies remain highly warranted in order to reach a better understanding of the potential interest of glycated albumin, oxidative stress, and glycoxidation parameters in the monitoring of type 2 diabetic patients. Nonetheless, the present study brings new insights on fluorescent AGEs as a putative indicator for vascular event prediction in diabetic patients.

## Figures and Tables

**Table 1:** Clinical and biochemical characteristics of the study participants.

	ND	D	
*N*	*n* = 31	*n* = 75	
Male Sex (%)	*n* = 9 (29.0)	*n* = 22 (29.3)	
With vascular complication	*n* = 0	*n* = 44	

	*Average +/− SD*	*Average +/− SD*	*p value*
Age	51.2 +/− 17.7	59.1 +/− 10.3	0.0164
BMI		30.2 +/− 5.5	
Total protein (g/L)	69.8 +/− 6.3	70.6 +/− 4.5	0.5201
Albumin (g/L)	40.5 +/− 7.1	42.3 +/− 2.8	<0.0001
Fructosamine (mol/g)	2.67 +/− 0.41	4.78 +/− 0.77	<0.0001
HbA1c (%)	5.3 +/− 0.43	9.1 +/− 1.1	<0.0001
Total cholesterol (mmol/L)	4.4 +/− 0.82	4.14 +/− 1.04	0.2488
HDL-cholesterol (mmol/L)	1.27 +/− 0.44	1.12 +/− 0.29	0.0771
LDL-cholesterol (mmol/L)	2.82 +/− 0.75	2.50 +/− 0.90	0.1079
Triglycerides (mmol)	1.42 (0.99–1.78)	1.26 (0.98–1.66)	0.67
(25–75th percentile)			
ApoA (mmol/L)	1.42 +/− 0.37	1.30 +/− 0.22	0.0807
ApoB (mmol/L)	0.84 +/− 0.16	0.89 +/− 0.25	0.3291

Study participants are divided into two groups: diabetic (D, *n* = 75) and nondiabetic (ND, *n* = 31) patients.

**Table 2:** Correlation coefficients between glycation, oxidation, and glycoxidation biomarkers for the total panel.

	Thiols (*µ*mol/g)				
Thiols (*µ*mol/g)	—				
		IMA/ALB (g/L)			
IMA/ALB (g/L)	0.186	—			
			Fluo-AGE (AU)		
Fluo-AGE (AU)	0.015	0.219^*∗*^	—		
				AGE (ng/mL)	
AGE (ng/mL)	0.017	0.048	0.028	—	
					GA (mg/L)
GA (mg/L)	0.183	0.302^*∗∗∗*^	0.036	0.399^*∗∗∗*^	—
Fructosamines (mol/g)	0.130	0.430^*∗∗∗*^	0.032	0.135	0.492^*∗∗∗*^

Glycation parameters: fructosamine, glycated albumin (GA); oxidation parameters: ischemia-modified albumin (IMA), thiols; glycoxidation parameters: advanced glycation end-products (AGE), fluorescent advanced glycation end-products (Fluo-AGE). Total panel includes diabetic and nondiabetic patients (*N* = 106). Univariate correlation coefficients and significance between different biochemical parameter values compared by peer were calculated according to Pearson's method: ^*∗*^*p* < 0.05, ^*∗∗∗*^*p* < 0.001.

**Table 3:** Clinical and biochemical characteristics of the diabetic patients.

	NVD	VD	
*N*	*n* = 31	*n* = 44	
Male Sex (%)	*n* = 7 (22.6)	*n* = 15 (34.1)	*p* = 0.270

	*Average +/− SD*	*Average +/− SD*	*p value*
Age	56.2 +/− 2.3	60.8 +/− 1.4	0.070
BMI	31.0 +/−1.3	29.7 +/− 0.8	0.036
Total protein (g/L)	71.1 +/− 4.8	70.3 +/− 4.4	0.369
Albuminemia (g/L)	41.9 +/− 2.8	42.5 +/− 2.8	0.156
Fructosamine (mol/g)	4.68 +/− 0.78	4.84 +/− 0.77	0.392
HbA1c (%)	8.9 +/− 0.2	9.1 +/− 0.2	0.31
Glycemia (mmol/L)	9.0 +/− 2.9	9.2 +/− 3.1	0.754
Total cholesterol (mmol/L)	4.17 +/− 0.84	4.11 +/− 1.17	0.807
HDL-cholesterol (mmol/L)	1.13 +/− 0.30	1.12 +/− 0.28	0.882
LDL-cholesterol (mmol/L)	2.49 +/− 0.79	2.52 +/− 0.97	0.887
Triglycerides (mmol)	1.34 (0.99–1.77)	1.49 (0.99–1.91)	0.520
(25–75th percentile)			
ApoA (mmol/L)	1.30 +/− 0.22	1.30 +/− 0.22	1.000
ApoB (mmol/L)	0.89 +/− 0.21	0.88 +/− 0.28	0.866
Urine protein (g/L)	*⁡*0.09 (0.06–0.13)	0.10 (0.07–0.16)	0.370
(25–75th percentile)			
Microalbuminuria (mg/L)	63.1 +/− 153.2	83.5 +/− 192.5	0.606

The panel of diabetic patients is divided into two groups: diabetic patients with and without vascular disease (resp., VD, *n* = 44, and NVD, *n* = 31).

**Table 4:** Correlation coefficients between glycation, oxidation, and glycoxidation biomarkers in the diabetic group.

	Thiols (*µ*mol/g)				
Thiols (*µ*mol/g)	—				
		IMA/ALB (g/L)			
IMA/ALB (g/L)	0.074	—			
			Fluo-AGE (AU)		
Fluo-AGE (AU)	0.068	0.011	—		
				AGE (ng/mL)	
AGE (ng/mL)	0.072	0.027	0.036	—	
					GA (mg/L)
GA (mg/L)	0.061	0.174	0.103	0.4312^*∗∗∗*^	—
Fructosamines (mol/g)	0.151	0.181	0.012	0.110	0.230

Glycation parameters: fructosamine, glycated albumin (GA); oxidation parameters: ischemia-modified albumin (IMA), thiols; glycoxidation parameters: advanced glycation end-products (AGE), fluorescent advanced glycation end-products (Fluo-AGE). The panel includes diabetic patients with and without vascular disease (*N* = 75). Univariate correlation coefficients and significance between different biochemical parameter values compared by peer were calculated according to Pearson's method: ^*∗∗∗*^*p* < 0.001.

**Table 5:** Associations between fluorescent AGE levels and personal history of vascular disease in univariate and multivariate analyses (^*∗*^adjusted for age, sex, and BMI).

	Univariate odds ratio for vascular disease (IC 95%)	*p* value	Multivariate^*∗*^ odds ratio for vascular disease (IC 95%)	*p* value
Fluo AGE (per 1000 U)	1.32 (1.01–1.75)	0.04	1.35 (0.97–1.88)	0.07

## Abbreviations

ACB: Albumin-cobalt binding
AGE: Advanced glycation end-products
ACS: Acute coronary syndrome
BMI: Body mass index
CVD: Cardiovascular disease
CVA: Cerebrovascular accidents
Fluo-AGE: Fluorescent advanced glycation end-products
GA: Glycated albumin
IMA: Ischemia-modified albumin
MI: Myocardial infarction
nf-AGE: Nonfluorescent AGE
ROS: Reactive oxygen species
T2DM: Type 2 diabetes mellitus.
